# Contamination Rate of Cryopreserved Umbilical Cord Blood Is Inversely Correlated with Volume of Sample Collected and Is also Dependent on Delivery Mode

**DOI:** 10.1093/stcltm/szac020

**Published:** 2022-04-29

**Authors:** Susanne Reuther, Kathrin Floegel, Gunther Ceusters, Veronica Albertini, Jakub Baran, Wolfram Dempke

**Affiliations:** Eticur Germany GmbH, Munich, Germany; Ludwig-Maximillians University Munich, Munich, Germany; Eticur Germany GmbH, Munich, Germany; Eticur Germany GmbH, Munich, Germany; Famicord SA Suisse, Contone, Switzerland; Famicord SA Suisse, Contone, Switzerland; PBKM FamiCord Group, Warsaw, Poland; Ludwig-Maximillians University Munich, Munich, Germany; Worldwide Clinical Trials, Nottingham, United Kingdom

**Keywords:** umbilical cord blood, sample volume, contamination, bacterial strains, delivery mode

## Abstract

Cord blood (CB) collected at birth has become a valuable stem cell source for hematopoietic stem cell transplantation (HSCT). However, the collection of umbilical cord blood always bears a risk of microbiological contamination, both in vaginal birth and in cesarean section. A total of 10 054 umbilical cord stem cell samples were successfully cryopreserved between 2010 and 2020, of which 783 (8%) samples were tested positive for bacterial contamination. Umbilical CB with a volume of less than 60 mL showed a bacterial contamination rate of 12%, and above 60 mL volume a rate of 6% was found demonstrating an inverse relationship between sample volume and contamination rate (correlation coefficient *r* = −0.9). The contamination rate was associated with the mode of delivery and showed a significantly higher contamination rate of 9.7% when compared with cesarean deliveries (1.4%). The 10-year period consistently shows an average contamination rate between 4% and 6% per year. It is conceivable that the inverse relationship between volume and contamination rate might be related to thinner veins although no scientific evidence has been provided so far. The lower contamination rate in cesarean sections appears to be related to the sterile operating setting. Overall, the rate of bacterial contamination varies and depends on the type of birth, the way of delivery, and probably the experience of the staff.

## Graphical Abstract

**Figure:**
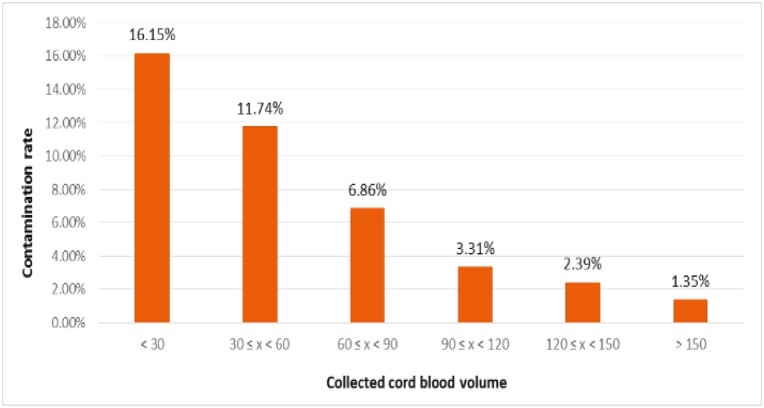

## Introduction

Umbilical cord blood (UCB) stem cells have been established as an alternative stem cell source for pediatric and adult patients with various oncologic, hematologic, immunologic, and inherited metabolic disorders, especially by lacking a related or unrelated donor.^1-3^ Cord blood is also a very attractive alternative stem cell source because of the increased level of HLA disparity that can be tolerated. This is of particular importance for patients from racial and ethnic minorities, as for those it can be difficult to find a donor.^1,4^.

Numerous retrospective studies from recent years have shown that UCB transplantation in patients with hematological malignancies can result in disease-free survival (DFS) comparable to that of adult donor transplants.^5,6^ In addition, few studies have confirmed that the relapse rate after UCB transplantation is low compared to unrelated donor transplants, indicating that UCB may be the preferred source for patients at high risk of relapse.^7^ UCB transplantation has the advantage of a lower rate of chronic graft-versus-host disease (GvHD) compared to hematopoietic stem cells from peripheral blood, which is reflected in lower long-term morbidity and mortality.^7^ Research studies have shown that cord blood transplants can also be performed in cases even when the donor and the recipient are partially matched, so cord blood increases the patient’s chance to find a more suitable donor.^8,9^ However, with increasing differences in the donor and recipient HLA systems, the risk of delayed or absent hematopoietic reconstitution also increases in UCB transplantation.^8,10^ In contrast, the greater availability of high-quality and high-cell-content UCB units has resulted in increasingly improved engraftment and survival outcomes after UCB transplantation.^11,12^

There are various providers worldwide, which accompany or sometimes even offer the process from collection to storage. Importantly, the collection of UCB always bears a risk of microbiological contamination, both in spontaneous/vaginal birth and in cesarean section.^13^ Cord blood preparations with latent virus and environmental toxin contaminations are lower than those with a bone marrow transplant, but appear to be more highly contaminated by bacteria than those from bone marrow and peripheral blood are, which may be due to the collection technique.^14-16^ Bacterial contamination of human blood products is another challenge as it occurs quite frequently and it is observed that bacteria developed resistance, which can be life-threatening, especially in immune-compromised patients.^17,18^ Therefore, sterility testing is mandatory according to the guideline standards to prevent transplantation transmitted bacterial infections causing severe transfusion-associated sepsis in immune-suppressed patients.^19,20^

Moreover, bacterial contamination of hematopoietic stem cells obtained from UCB may cause a rejection of a stem cell graft.^21^ In case of contamination, a bacterial determination should be carried out again from a suitable reference sample to exclude second contamination or higher risk of unsuccessful clinical outcome, so high quality and safety of hematopoietic stem cells are required for any successful transplantation.^16^ Using sterile techniques and a closed system during the whole preparation process is usually used to reduce the risk of any bacterial contamination.^8,16^ Freezing bags or tubes must comply with the current state of technology and exclude cross-contamination between different samples during storage.^16^

The aim of this study was to investigate the bacterial contamination rate related to the delivery mode including collection experiences of the staff and collection sample volume. In addition, the most commonly detected bacterial strains were determined.

## Methods

A total of 10 054 UCB samples for potential autologous use was cryopreserved in the transfusion medicine and hemostasis department from September 2010 to September 2020 in cooperation with the Erlangen University Hospital (Germany).

Institutions that manufacture, test, process, store, or market stem cell products in Germany must ensure compliance with legal requirements, maintain a quality management system in accordance with Section 3 AMWHV (“Arzneimittel- und Wirkstoffherstellungs-verordnung”) and the principles of “good manufacturing practice” (EU GMP Guideline). The UCB collection was carried out in all maternity clinics in Germany that had signed a contract with the Eticur GmbH Germany company in accordance with the current SOP “Guidelines for Transplantation of Stem Cells Part III, Umbilical Cord Stem Cells, 3.1 Collection” and the individual preparations were carried out by the transfusion medicine and hemostasis department of the University Hospital Erlangen in accordance with the SOP “Guidelines for Transplantation of Stem Cells, Part III Umbilical Cord Stem Cells, 4th CB Stem Cell Preparation.” The University Hospital of Erlangen is licensed by the Federal Agency PEI (Paul Ehrlich Institute). Eticur GmbH Germany is a 100% subsidiary of Famicord Europe and has established collaboration with the University Hospital Erlangen. In this regard, the applied methods for cryopreservation and microbiology testing were identified and described in SOPs (validated according to Ph.Eur. section 2.6.27).

Informed consent from the parents, a prescribed anamnesis questionnaire, and a production protocol were mandatory for the collection of UCB. All clinics and the University Hospital Erlangen provided a valid manufacturing permit. Approval for the pre-drug stage was carried out by the “Qualified Person” in accordance with § 14 AMG (German Medical Law).

Cord blood samples were transported immediately after birth to the stem cell laboratory were analyzed and cryopreserved (10% v/v DMSO) within 48 h from the delivery time. Every sample was analyzed in terms of total blood volume, a total count of CD34^+^ cells, total nucleated cell (TNC, CD45^+^) count, and colony-forming units (CFU). The CFU method was performed according to the protocol described by Dempke et al,^22^ which shows the in vitro capacity of the proliferation of hematopoietic stem cells.

CD34^+^ and CD45^+^ cells were analyzed by immunofluorescence on FACSCalibur (Becton Dickenson, Heidelberg) using anti-CD34-PE and anti-CD45-FITC (Becton Dickenson, Heidelberg) according to the protocol described by Cassens et al.^23^

To check the quality parameters such as the microbiological control, the purity, and the vitality of the product as well as to control the manufacturing process or cryopreservation, sufficient preparation samples according to Section 18 (1) AMWHV must be ensured until clinical use. These samples must be stored under conditions comparable to those of the umbilical cord blood stem cells.

In an attempt to determine the contamination with bacteria samples were taken from the incoming cord blood bag as well as the processed stem cell preparation followed by microbiological cultures were performed by using BD Bactec Standard Anaerobic/F 40 mL culture vials (REF 442024) and BD Bactec Standard/10 Aerobic/F 40 mL culture vials (REF442027) (Becton Dickenson, BD, Heidelberg) to detect anaerobic and aerobic bacteria after 7 incubation days. The sample volume was identical for all samples (percentage volume). The method validation included a slow grower and all standard bacteria and fungi. Each bag was tested twice and both assays (primary and secondary contamination rates) were comparable (data not shown).

## Results

**Figure 1.**
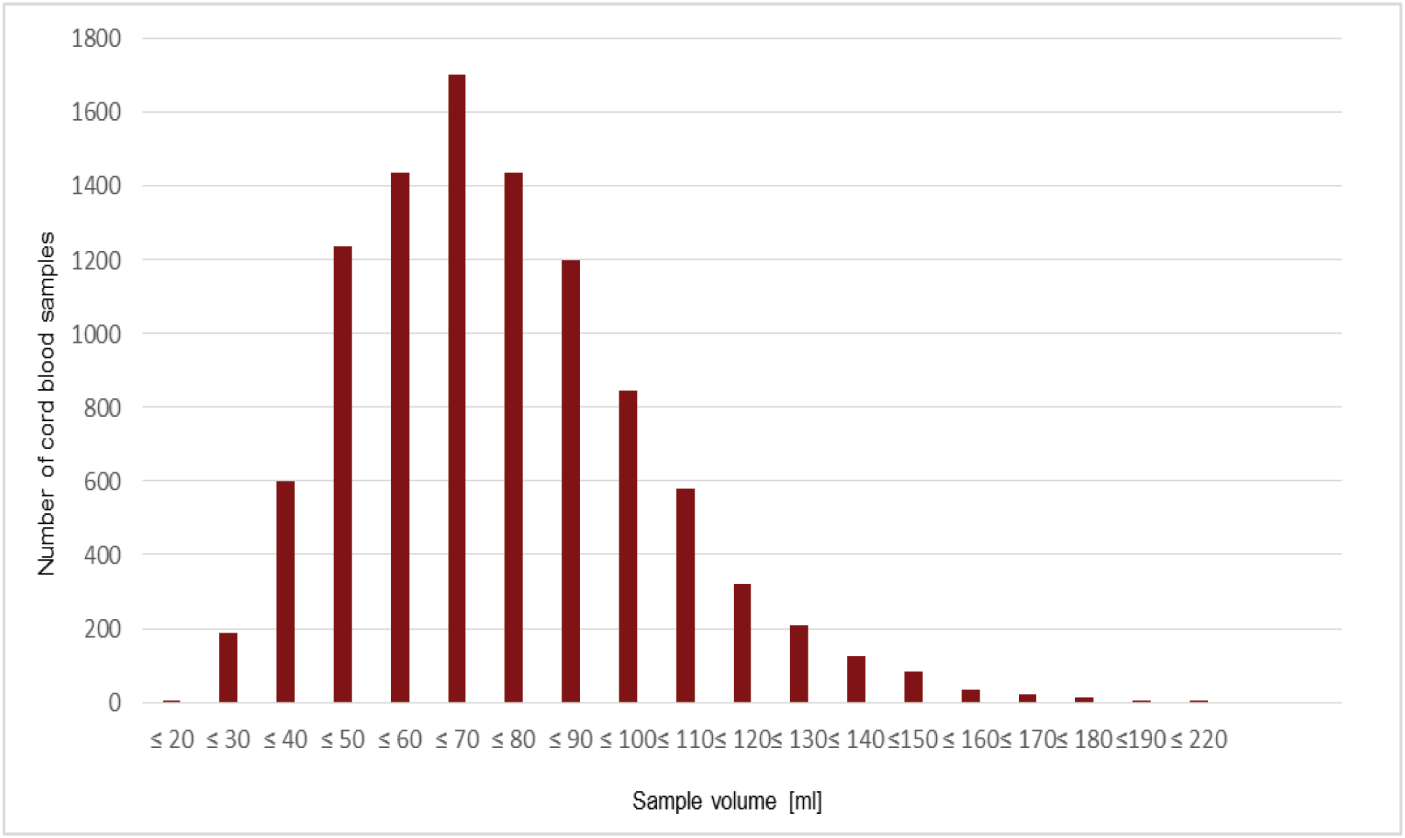
Number of cord blood samples related to sample volume (mL) showing a Gaussian-like distribution.

A total of 10 054 UCB stem cell samples were successfully cryopreserved between September 2010 and September 2020. Most cord blood samples had volumes between 50 and 100 mL. The overall distribution of all cord blood samples taken shows a Gaussian-like distribution with the maximum at less than or equal to 70 mL volume (Fig. 1).

**Figure 2.**
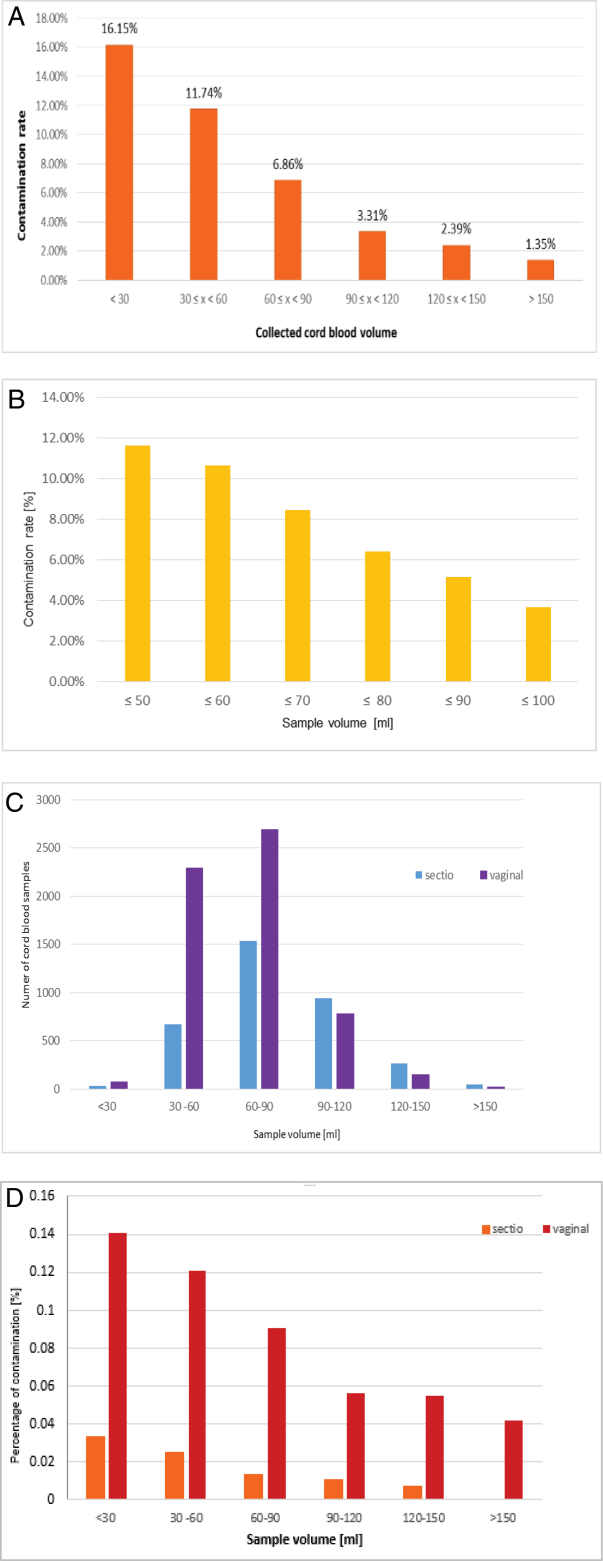
Contamination rate (%) in correlation with cord blood volume, correlation coefficient *r* = −0.9 **(A**), in correlation of all cord blood samples with a volume between 50 and 100 mL **(B)**. Number of cord blood samples in correlation to sample volume (mL) and delivery mode **(C**) and related to delivery mode and collection sample volume **(D)**.

From 10 054, 783 (7.8%) samples were tested positive for bacterial contamination. UCB with a volume of less than 60 mL showed a contamination rate of 12%, and above 60 mL volume, a rate of 6% was found demonstrating an inverse relationship between sample volume and contamination rate (correlation coefficient *r* = −-0.9) (Fig. 2A).

Considering all samples with a volume of 50–100 mL, an inverse correlation between sample volume and contamination rate is clearly shown, the higher the volume collected, the lower the contamination rate (Fig. 2B**).**

Furthermore, the results show that vaginal birth is much more frequently performed than cesarean section, and a Gaussian-like distribution can also be seen for both birth modes, where a maximum is seen with the highest number of collections is at a sample volume of 60–90 mL (Fig. 2C).

Regarding the contamination rate related to collection sample volume as well as delivery mode, both birth modes showed the inverse correlation between sample volume and contamination rate (Fig. 2D). For spontaneous birth, depending on the volume, the contamination rate ranges from 14.1% for less than 30 mL to 4.17% for more than 150 mL of cord blood collected. In contrast, cesarean sections showed a contamination rate between 3.3% for less than 30 mL and 0% for more than 150 mL volume (Table 1).

**Table 1.** Contamination rate (%) related to delivery mode and collection sample volume.

**Cord blood collection volume (mL)**	**Cesarian section (%)**	**Vaginal delivery (%)**
<30	3.33	14.10
30-60	2.53	12.10
60-90	1.31	9.05
90-120	1.06	5.63
120-150	0.75	5.48
>150	0.00	4.17
Average of contamination rate	1.50	8.42

In addition, the contamination rate associated with the mode of delivery showed a significantly higher average contamination rate of 8.4% compared with cesarean deliveries with only 1.5% (Table 1; Fig. 2D).

**Figure 3.**
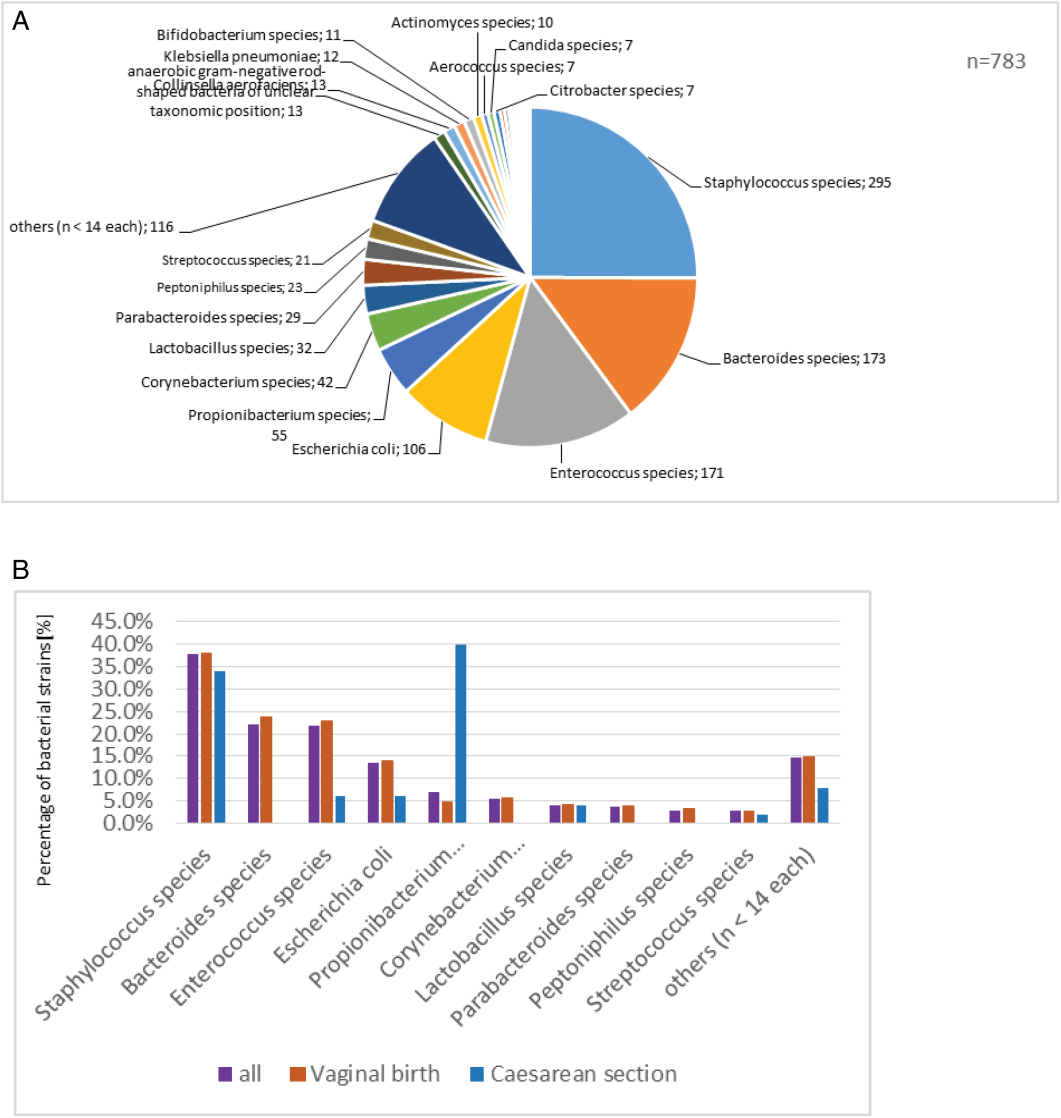
All detected bacterial strains in the 783 non-sterile samples (**A**). Detected percentage of bacterial strains in all cord blood samples and divided into vaginal birth and cesarean section (**B**).

Considering all 783 non-sterile samples the most detected bacterial strains were *Staphylococcus*- (37.7%), *Bacteroides*- (22.1%), and *Enterococcus*-strains (21.8%), as well as *Escherichia coli* species (13.5%) (Table 2A). All detected bacteria strains are shown in Fig. 3A. The 3 largest bacterial strains show the following subfamilies as the most identified: In the staphylococcus group, the *Staphylococcus epidermidis* subfamily is the most detected with 38.9%; *Bacteroides* reveal *Bacteroides vulgatus* as the most found subfamily with 43.3%. *Enterococcus faecalis* is the most determined in its family with 82.8%. No unexpected findings were found according to the standards of the European Pharmacopoeia (Table 2B*i–iii*).

**Table 2.** Most commonly detected bacterial strains in the 783 non-sterile samples (A) and the most detected bacterial strains in all non-sterile cord blood samples and the determined subfamilies (B*i*-*iii*).

**Bacterial strain**	**Number of cases**	**Percentage of bacterial strains [%]**
**(A) 783 non-sterile samples**		
*Staphylococcus* species	295	37.68
*Bacteroides* species	173	22.09
*Enterococcus* species	171	21.84
*Escherichia coli*	106	13.54
*Propionibacterium* species	55	7.02
*Corynebacterium* species	42	5.36
*Lactobacillus* species	32	4.09
*Parabacteroides* species	29	3.70
*Peptoniphilus* species	23	2.94
*Streptococcus* species	21	2.68
**(B *i* ) Staphylococcus subgroup**		
*Staphylococcus epidermidis*	115	
Coagulase-negative *Staphylococcus* species	102	
*Staphylococcus hominis*	27	
*Staphylococcus haemolyticus*	20	
*Staphylococcus capitis*	17	
*Staphylococcus lugdunensis*	5	
*Staphylococcus saccharolyticus*	2	
*Staphylococcus caprae*	1	
*Staphylococcus cohnii*	1	
*Staphylococcus condimenti*	1	
*Staphylococcus pettenkoferi*	1	
*Staphylococcus warneri*	1	
*Staphylococcus xylosus*	1	
*Stapyhlococcus capitis*	1	
	295 total	
**(B *ii*) Bacteroides subgroup**		
*Bacteroides vulgatus*	75	
*Bacteroides uniformis*	38	
*Bacteroides* species	14	
*Bacteroides fragilis*	12	
*Bacteroides ovatus*	10	
*Bacteroides stercoris*	6	
*Bacteroides thetaiotaomicron*	5	
*Bacteroides cellulosilyticus*	4	
*Bacteroides caccae*	3	
*Bacteroides nordii*	2	
*Bacteroides faecis*	1	
*Bacteroides hetaiotaomicron*	1	
*Bacteroides massiliensis*	1	
*Bacteroides salyersiae*	1	
	173 total	
**B (*iii*) Enterococcus subgroup**		
*Enterococcus faecalis*	99	
*Enterococcus species*	12	
*Enterococcus faecium*	7	
*Enterococcus avium*	1	
*Enterococcus durans*	1	
*Enterococcus hirae*	1	
	121 total	

**Figure 4.**
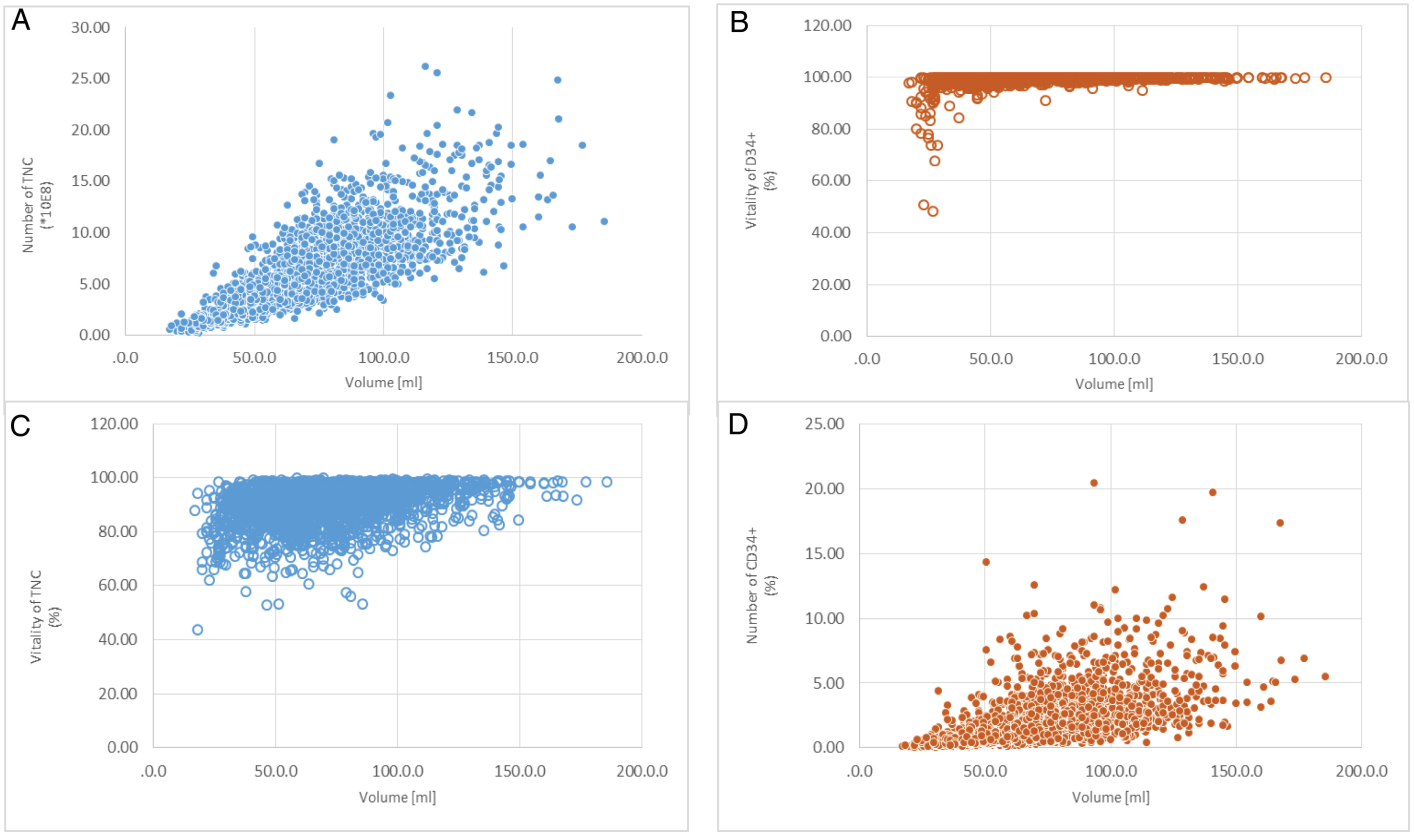
Number of TNC (**A**) and CD34^+^ stem cells (**B**) in correlation with the cord blood volume (mL). Vitality (%) of TNC (**C**) and vitality (%) CD34^+^ stem cells (**D**) both related to the sample volume respectively (mL).

At cesarean section, the most frequent strains in collected UCB is the propionibacterium acnes with 40% as well as 34% of *staphylococcus* species, 6% of *Enterococcus species*, and *E. coli* species with 6% were detected (Table 3; Fig. 3B**).** The quality of the umbilical cord stem cell preparation is also determined using the cell number of TNC (CD45^+^) as well as the CD34^+^ stem cells that are included in the TNC population. In addition, the viability of both cell populations is indicative of the quality of the stem cells. It is clearly shown that the higher the volume of the umbilical cord blood, the higher is the TNC cell count (Fig. 4A). Regarding the CD34^+^ stem cells, this correlation cannot be clearly identified. A clear range of variation is shown in Fig. 4B. A direct correlation between sample volumes and the vitality of TNC as well as CD34^+^ stem cells could not be established as the vitality of both cell populations does not primarily depend on the volume but also on the transport and storage conditions as well as on the processing of the cord blood into the stem cell preparation (internal quality data, not shown).

**Table 3.** Distribution of identified bacteria depended on the mode of delivery.

**Bacterial strain**	**Percentage of bacterial strains (%)**		
	**All cases (independent from delivery mode)**	**Vaginal delivery**	**Cesarean section**
*Staphylococcus* species	37.68	37.93	34.00
*Bacteroides* species	22.09	23.60	0.00
*Enterococcus* species	21.84	22.92	6.00
*Escherichia coli*	13.54	14.05	6.00
*Propionibacterium* species	7.02	4.77	40.00
*Corynebacterium* species	5.36	5.73	0.00
*Lactobacillus* species	4.09	4.09	4.00
*Parabacteroides* species	3.70	3.96	0.00
*Peptoniphilus* species	2.94	2.14	0.00
*Streptococcus* species	2.68	2.73	2.00

**Figure 5.**
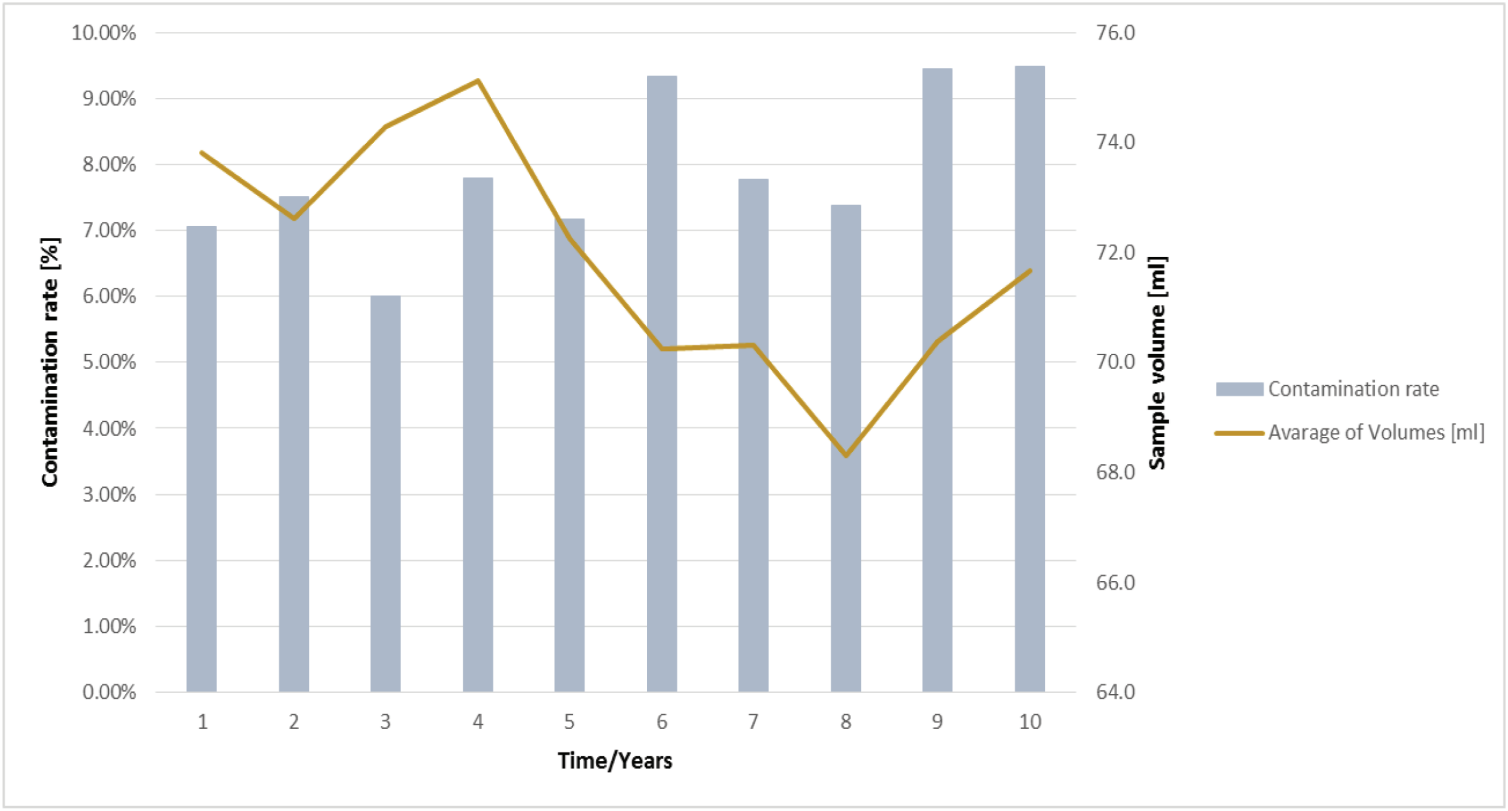
History of the contamination rate (%) per year shown for the last 10 years.

Regarding the TNC, it became apparent that a vitality of 70%-80% was tendentially achieved with smaller volumes, in contrast to the CD34^+^ stem cells, which (with a few exceptions) display a very good vitality of over 90% independent of the sample volume (Fig. 4C, 4D). Considering the 10-year period, a contamination rate between 6% and 9.5% per year was found (Fig. 5). Although it appears that the contamination rates are higher after 5 years, the differences are not significant (*P* > .1, Student *t*-test).

## Discussion

The main objective of this study was the investigation of bacterial contamination rate under a routine condition in the cord blood stem cell products after vaginal delivery and cesarean section related to sample volume and bacterial strains. Additionally, the correlation between the cell numbers, sample volume, and delivery mode has been established.

The quality and associated safety of hematopoietic stem cells are one of the most important requirements for successful transplantation. Another important factor is considered the high concentration of adult pluripotent progenitor stem cells with increased proliferative potency, which are usually not yet affected by environmental factors and are young and vital. It is possible that due to the relative immaturity of the T cells in the CB, greater immunological tolerance can be expected after transplantation.^2,24^ This is probably one reason for fewer cases of acute or chronic GvHD. It has also been reported that not all HLA characteristics need to be matched in allogeneic hematopoietic stem cell transplantation, making cord blood an alternative to bone marrow or peripheral blood stem cell transplantation.^3,8,9,24-27^

In our study, 10 054 UCB stem cell preparations were cryopreserved between 2010 and 2020, with all volumes showing a Gaussian normal distribution. Thereby, most of the UCB samples have a volume of 50-100 mL and peaked at approximately 70 mL (Fig. 1), whereas according to the applicable standard operation procedure (SOP) a minimum volume of at least 60 mL had to be collected. Although numerous samples contained less blood volume, storage was still requested after the parents had become aware of the reduction in quality, provided that the parents had been informed of the reduction in quality beforehand, which is required by the transfusion law. Although the TNC cell concentration is proportional to the blood volume (Fig. 4A), this is not the same for the CD34^+^ stem cells (Fig. 4B), so that the storage of smaller volumes is also acceptable. In general, a minimum concentration of ≥2.5 × 10^8^ TNC and of ≥1.0 × 10^6^ of CD34^+^ stem cells with a respective viability of ≥90% and ≥95% was required, in accordance with the German stem cell guideline current at the time.^28^

No difference was found between vaginal birth and cesarean section in terms of obtained cord blood sample volumes. This is in line with the results of other studies.^29,30^ In contrast, other authors report that more blood volume was collected during cesarean section than during vaginal birth.^31,32^ Moreover, no difference between the birth modes in the cell concentrations of TNC and CD34^+^ stem cells were found, consistent with the reports of other researchers.^30,33^ However, it contradicts the statements of some researchers who found a higher CD34^+^ stem cell concentration at vaginal birth or cesarean section.^32,34^ Mancinelli et al^32^ postulated that in spontaneous deliveries an increase in CD34^+^ cells due to the narrow birth canal, as pressure is applied to the thorax and abdomen of the child and this pressure allows more cells to circulate compared to cesarean deliveries.^35^ In contrast, Yamada et al^34^ confirmed a larger volume and higher content of CD34^+^ cells in collections from cesarean sections.^34^ It was hypothesized that the difference was due to the position of the umbilical cord and the infant before clamping. When the newborn is placed on the maternal abdomen after delivery, the volume and content of CD34^+^ cells in the cord blood increases.^34,36^

Some reports also described that vaginal delivery was associated with higher TNC counts than at cesarean section.^28,37,38^ In agreement with Platz et al^13^ their study showed that cord blood collections may have comparable amounts of necessary cells in both vaginal births and cesarean sections.^13^ Additionally, it is also hypothesized that the causes of the lower cell content in primary cesarean sections can be attributed to for instance preoperative hemodilution.^13^ These results of contamination rates in this study are well within the range of those values reported in the literature (0.9%-8.6%).^18,39-44^

The inverse relationship between volume and contamination rate might be related to small veins, especially for smaller volumes. Small veins may result in a longer collection time, which may have some (albeit limited) impact on the complete sterility of the cord throughout the collection process. Furthermore, it should be noted that inexperienced staff may also contribute to higher contamination rates. Although this was not systematically evaluated in our study, there was a trend to a higher contamination rate when blood samples were drawn by less experienced staff and a separate study is underway to further address this observation.

Also, re-attempting collection, especially if the umbilical cord collapses quickly, can sometimes occur and sterility may no longer be optimal. Beside the vaginal or as perianal microbiomes source for contamination, kit contamination as well as bacteria from skin cannot be excluded as source during collection preparation and cryopreservation.^15,16^ Moreover, it is evident that the preparation of CB for clinical use is another challenge due to the small sample volume and special sterility requirements that must be met. Studies are currently ongoing to optimize UCB sample collection with a focus on diluting the starting material, determining the best time for sample collection, and collecting test samples from UCB residues, ie, red blood cells.^45^ However, it should be noted that any sampling for testing would reduce the quantity of stem cells available for transplantation.

Further results regarding the contamination rate related to collection sample volume as well as delivery mode, both birth modes showed also the inverse correlation between sample volume and contamination rate (Fig. 2D). For spontaneous birth, depending on the volume, the contamination rate ranges from 14.1% for less than 30 mL to 4.17% for more than 150 mL of cord blood collected. In contrast, cesarean sections showed a contamination rate between 3.3% for less than 30 mL and 0% for more than 150 mL volume (Table 1).

The dependence of contamination rate on the mode of delivery is even more evident in the significantly higher average contamination rate of 8.4% for vaginal deliveries compared to 1.5% for cesarean deliveries (Table 1; Fig. 2D). The relation of the results to each other is comparable to the one described here, but the contamination rates in this study are higher than other authors already showed contamination rates of 4.1%, 5.31%, 5.6% at vaginal birth and 0.79% and 0.64% from cesarean sections are shown.^13,21,39^ The lower contamination rate in cesarean sections is caused by the environmental conditions of an operating room.^13,21^

In all non-sterile cord blood stem cell products (*n* = 783) the most detected bacterial strains were *Staphylococcus* with 37.7% and *Bacteroides* species with 22.1% as well as *E. coli* species with 13.5% (Fig. 3A; Table 2A) corresponding to results of other authors.^14-17,42,44,46^ As bacterial subfamilies *S. epidermidis* (38.9%, *n* = 295) and *Bacteroides vulgatus* (43.4%, *n* = 173) were found (Table 2B*i* and *ii***).** The third most common bacterial strain identified was *Enterococcus* (21.8%, *n* = 121), especially *Enterococcus faecalis* as the most common with 81.8% (Table 2Biii). The results are comparable with those from other authors.^16,18,21^

The most frequently described bacterial strain *Staphylococcus* species identified in cord blood samples from vaginal births was also found in cesarean section samples, but it is remarkable that propionibacterium acnes was the most frequently detected with 40% (*n* = 50) in cesarean sections (Fig. 3B; Table 3).

Other authors also confirm the identification of relatively high concentrations of propionibacterium acnes in cord blood samples.^20,44,47^ Even if the average bacterial contamination rates is not higher than those of other studies, the analysis of the last 10 years shows an average contamination rate between 6% and 9.5% (Fig. 5), which are comparable to the described values from the literature.^16^

Even if not all bacteria survive the process of cryopreservation,^44^ it is clear the processes starting with the collection of the umbilical cord blood, through the processing to the stem cell preparation to the cryopreservation according to the national guidelines and laws must be trained and optimized repeatedly to be able to fulfill the required parameters.^14^ Furthermore, it must be ensured that multi-resistant bacteria, one of the great challenges of our time, do not continue to advance.

## Conclusion

The contamination rate of cryopreserved UCB is dependent on delivery mode and is inversely correlated with the volume of sample collected at birth. Based on the study presented here several lines of evidence indicate that bacterial contamination rates of UCB collected at birth for transplantation purposes may be reduced by the collection of samples after cesarean delivery together with a high sample volume. In addition, utilizing experienced and well-trained collection staff is able to successfully complete the collection in a sterile manner even in the presence of smaller veins, which can successfully reduce insufficient puncture maneuver, which can clearly increase the contamination rate.

## Data Availability

The data that supports the findings of this study are available from the corresponding author upon reasonable request.

## References

[1] Barker JN , Weisdorf DJ, DeFor TE, et al. Transplantation of 2 partially HLA matched umbilical cord blood units to enhance engraftment in adults with hematologic malignancy. Blood. 2005;105(3):1343-1347. 10.1182/blood-2004-07-2717. 15466923

[2] Ballen K. Update on umbilical cord blood transplantation. F1000Res. 2017; 6(24):1556. 10.12688/f1000research.11952.1. 28928957 PMC5580430

[3] Gupta AO , Wagner JE. Umbilical cord blood transplants: current status and evolving therapies. Front Pediatr. 2020;8(2):570282. 10.3389/fped.2020.570282. 33123504 PMC7567024

[4] Gragert L , Eapen M, Williams E, et al. HLA match likelihoods for hematopoietic stem-cell grafts in the U.S. registry. N Engl J Med. 2014;371(4):339-348. 25054717 10.1056/NEJMsa1311707 PMC5965695

[5] Brunstein CG , Gutman JA, Weisdorf DJ, et al. Allogeneic hematopoietic cell transplantation for hematologic malignancy: relative risks and benefits of double umbilical cord blood. Blood. 2010;116(22):4693-4699. 20686119 10.1182/blood-2010-05-285304 PMC2996124

[6] Ruggeri A , Labopin M, Sanz G, et al. Comparison of outcomes after unrelated cord blood and unmanipulated haploidentical stem cell transplantation in adults with acute leukemia. Leukemia. 2015;29(9):1891-1900. 25882700 10.1038/leu.2015.98

[7] Milano F , Gooley T, Wood B, et al. Cord-blood transplantation in patients with minimal residual disease. N Engl J Med. 2016;375(10):944-953. 27602666 10.1056/NEJMoa1602074 PMC5513721

[8] Sivakumaran N , Rathnayaka I, Shabbir R, et al. Umbilical cord blood banking and its therapeutic uses. INt J Scie Rese Inno Tech. 2018;5(1):160-170.

[9] Xue E , Milano F. Are we underutilizing bone marrow and cord blood? Review of their role and potential in the era of cellular therapies. F1000Res. 2020; 9:F1000 Faculty Rev-26. 10.12688/f1000research.20605.1 PMC6970216 31984133

[10] Maslova O , Novak M, Kruzliak P. Umbilical cord tissue-derived cells as therapeutic agents. Stem Cells Int. 2015; 2015:Article ID 150609. 10.1155/2015/150609. PMC4515303 26246808

[11] Dehn J , Spellman S, Hurley CK, et al. Selection of unrelated donors and cord blood units for hematopoietic cell transplantation: guidelines from the NMDP/CIBMTR. Blood. 2019;134(19):924-934. 10.1182/blood.2019001212. 31292117 PMC6753623

[12] Hough R , Danby R, Russell N, et al. Recommendations for a standard UK approach to incorporating umbilical cord blood into clinical transplantation practice: an update on cord blood unit selection, donor selection algorithms and conditioning protocols. Br J Haematol. 2016;172(3):360-370. 10.1111/bjh.13802. 26577457

[13] Platz A , Müller R, Aurich AC, et al. Gewinnung von Nabelschnurblut zur allogenen Stammzelltransplantation nach Spontangeburt und Sectio caesarea—Qualitätsparameter im Vergleich. Geburtshilfe und Frauenheilkunde. 2014;74.

[14] Clark P , Trickett A, Stark D, et al. Factors affecting microbial contamination rate of cord blood collected for transplantation. Transfusion. 2011;52(8):1770-1777. 10.1111/j.1537-2995.2011.03507.x. 22211719

[15] França L , Simões C, Taborda M, et al. Microbial contaminants of cord blood units identified by 16S rRNA sequencing and by API test system, and antibiotic sensitivity profiling. PLoS One. 2015;10(10):e0141152. 26512991 10.1371/journal.pone.0141152 PMC4626235

[16] Antoniewicz-Papis J , Lachert E, Rosiek A, et al. Microbial contamination risk in hematopoietic stem cell products: retrospective analysis of 1996-2016 data. Acta Haematol Pol. 2020;51(3):29-33.

[17] Domanović D , Cassini A, Bekeredjian-Ding I, et al. Prioritizing of bacterial infections transmitted through substances of human origin in Europe. Transfusion. 2017;57(5):1311-1317. 10.1111/trf.14036. 28236291

[18] Bello-López JM , Noguerón-Silva J, Castañeda-Sánchez JI, et al. Molecular characterization of microbial contaminants isolated from Umbilical Cord Blood Units for transplant. Braz J Infect Dis. 2015;19(6):571-577. 10.1016/j.bjid.2015.07.005. 26361843 PMC9425361

[19] Linder K , McDonald P, Kauffman C, et al. Infectious complications after umbilical cord blood transplantation for hematological malignancy. Open Forum Infect Dis. 2019;6:1-8. 10.1093/ofid/ofz037. PMC6386816 30815505

[20] Honohan A , Olthuis H, Bernards AT, et al. Microbial contamination of cord blood stem cells. Vox Sang. 2002;82(1):32-38. 10.1046/j.1423-0410.2002.00133.x. 11856465

[21] Ibrahim M , AL-Hajali S, Abdelmeguid M, et al. Contamination rates by delivery method of human umbilical cord blood samples in the United Arab Emirates and Gulf Cooperation Council Countries. Int J Stem Cell Res Ther. 2020;7(5):067. 10.23937/2469-570X/1410067.

[22] Dempke W , Nehls P, Wandl U, et al. Increased cytotoxicity of 1-(2-chloroethyl)-1-nitroso-3(4-methyl)-cyclohexylurea by pretreatment with O6-methylguanine in resistant but not in sensitive human melanoma cells. J Cancer Res Clin Oncol. 1987;113(4):387-391. 10.1007/bf00397725. 3597524 PMC12248309

[23] Cassens U , Fischer J, Fritsch G, et al. Auswertung der Analyse, Befunddarstellung und Dokumentation. Infusions Ther Transfusions Med. 1996;23(suppl 2):16-18.

[24] Montgomery FU , Cichutek K, Scriba PC et al. Richtlinie zur Herstellung und Anwendung von hämatopoetischen Stammzellzubereitungen—Erste Fortschreibung. Deutsches Ärzteblatt. | 20.02.2019 | 10.3238/arztebl.2019.rl_haematop_sz02.

[25] Nunes RD , Zandavalli FM. Association between maternal and fetal factors and quality of cord blood as a source of stem cells. Rev Bras Hematol Hemoter. 2015;37(1):38-42. 10.1016/j.bjhh.2014.07.023. 25638766 PMC4318845

[26] Grieco D , Lacetera N, Macis M, et al. Motivating cord blood donation with information and behavioral nudges. Sci Rep. 2018;8(1):252-264. 29321654 10.1038/s41598-017-18679-y PMC5762860

[27] Komanduri KV , St John LS, de Lima M, et al. Delayed immune reconstitution after cord blood transplantation is characterized by impaired thymopoiesis and late memory T-cell skewing. Blood. 2007;110(13):4543-4551. 10.1182/blood-2007-05-092130. 17671230 PMC2234787

[28] Jan RH , Wen SH, Shyr MH, et al. Impact of maternal and neonatal factors on CD34+ cell count, total nucleated cells, and volume of cord blood. Pediatr Transplant. 2008;12(8):868-873. 10.1111/j.1399-3046.2008.00932.x. 18643913

[29] Solves P , Fillol M, López M, et al. Mode of collection does not influence haematopoietic content of umbilical cord blood units from caesarean deliveries. Gynecol Obstet Invest. 2006;61(1):34-39. 10.1159/000088340. 16166778

[30] Sparrow RL , Cauchi JA, Ramadi LT, et al. Influence of mode of birth and collection on WBC yields of umbilical cord blood units. Transfusion. 2002;2(2):210-215. 10.1046/j.1537-2995.2002.00028.x. 11896337

[31] Aufderhaar U , Holzgreve W, Danzer E, et al. The impact of intrapartum factors on umbilical cord blood stem cell banking. J Perinat Med. 2003;31(4):317-322. 10.1515/jpm.2003.045. 12951888

[32] Mancinelli F , Tamburini A, Spagnoli A, et al. Optimizing umbilical cord blood collection: impact of obstetric factors versus quality of cord blood units. Transplant Proc. 2006;38(4):1174-1176. 16757298 10.1016/j.transproceed.2006.03.052

[33] Solves P , Perales A, Moraga R, et al. Maternal, neonatal and collection factors influencing the haematopoietic content of cord blood units. Acta Haematol. 2005;113(4):241-246. 10.1159/000084677. 15983430

[34] Yamada T , Okamoto Y, Kasamatsu H, et al. Factors affecting the volume of umbilical cord blood collections. Acta Obstet Gynecol Scand. 2000;79(10):830-833. 10.1034/j.1600-0412.2000.079010830.x. 11304964

[35] Kamble R , Pant S, Selby GB, et al. Microbial contamination of hematopoietic progenitor cell grafts-incidence, clinical outcome, and cost-effectiveness: an analysis of 735 grafts. Transfusion. 2005;45(6):874-878. 15934984 10.1111/j.1537-2995.2005.04178.x

[36] Grisaru D , Deutsch V, Pick M, et al. Placing the newborn on the maternal abdomen after delivery increases the volume and CD34+ cell content in the umbilical cord blood collected: an old maneuver with new applications. Am J Obstet Gynecol. 1999;180(5):1240-1243. 10.1016/s0002-9378(99)70623-x. 10329884

[37] Wen SH , Zhao WL, Lin PY, et al. Associations among birth weight, placental weight, gestational period and product quality indicators of umbilical cord blood units. Transfus Apher Sci. 2012;46(1):39-45. 22206793 10.1016/j.transci.2011.10.031

[38] Al-Sweedan SA , Musalam L, Obeidat B. Factors predicting the hematopoietic stem cells content of the umbilical cord blood. Transfus Apher Sci. 2013;48(2):247-252. 10.1016/j.transci.2013.01.003. 23415410

[39] Park JS , Shin S, Yoon JH, et al. Microbial contamination of donated umbilical cord blood. Ann Clin Microbiol. 2013;16(1):39.

[40] Donmez A , Aydemir S, Arik B, et al. Risk factors for microbial contamination of peripheral blood stem cell products. Transfusion. 2012;52(4):777-781. 10.1111/j.1537-2995.2011.03359.x. 21981571

[41] Meyer TPH , Hofmann B, Zaisserer J, et al. Analysis and cryopreservation of hematopoietic stem and progenitor cells from umbilical cord blood. Cytotherapy. 2006;8(3):265-276. 10.1080/14653240600735685. 16793735

[42] Anderson S. Retrieval of placental blood from the umbilical vein to determine volume, sterility, and presence of clot formation. Arch Pediatr Adolesc Med. 1992;146(1):36. 10.1001/archpedi.1992.0216013. 1736646

[43] Eichler H , Schaible T, Richter E, et al. Cord blood as a source of autologous RBCs for transfusion to preterm infants. Transfusion. 2000;40(9):1111-1117. 10988315 10.1046/j.1537-2995.2000.40091111.x

[44] Clark P , Tricket A, Saffo S, et al. Effects of cryopreservation on microbial-contaminated cord blood. Transfusion. 2013;54(3):532-540. 10.1111/trf.12323. 23808601

[45] Girard M , Laforce-Lavoie A, de Grandmont MJ, et al. Optimization of cord blood unit sterility testing: impact of dilution, analysis delay, and inhibitory substances. Transfusion. 2017;57(8):1956-1967. 10.1111/trf.14147. 28474347

[46] Cassens U , Ahlke C, Garritsen H, et al. Processing of peripheral blood progenitor cell components in improved clean areas does not reduce the rate of microbial contamination. Transfusion. 2002;42(1):10-17. 10.1046/j.1537-2995.2002.00013.x. 11896307

[47] Li M , Lim D, Tang KF, et al. Microbial contamination in umbilical cord blood: a comparison before and after cryopreservation. Stem Cells Transl Med. 2018;7(Suppl 1):S2-S2. 10.1002/sctm.12354.

